# Synthesis and characterization of new polyimides from diphenylpyrene dianhydride and *ortho* methyl substituted diamines

**DOI:** 10.1039/c8ra05991h

**Published:** 2018-09-12

**Authors:** R. Sulub-Sulub, M. I. Loría-Bastarrachea, J. L. Santiago-García, M. Aguilar-Vega

**Affiliations:** Unidad de Materiales, Centro de Investigación Científica de Yucatán A. C., Calle 43 No. 130, Chuburná de Hidalgo, C. P. 97200 Mérida Yucatán México mjav@cicy.mx

## Abstract

Three new polyimides were synthesized *via* one-step polycondensation from 3,8-diphenylpyrene-1,2,6,7-tetracarboxylic dianhydride (DPPD) with two diamines with *ortho* methyl substitution (MBDAM and HFI) and one diamine without *ortho* substituents (BAPHF). The effect of diamine structure in DPPD based polyimides' physical, thermal, mechanical and gas transport properties has been studied. The polyimide structure was confirmed by FTIR and ^1^H NMR. All polymers show high thermal stability with decomposition temperatures above 493 °C, and glass transition temperatures above 336 °C. Changes in packing density of polyimide membranes were assessed by wide angle X-ray diffraction and correlated to fractional free volume FFV. Polyimides based on rigid DPPD dianhydride exhibited an improved gas permeability and selectivity when *ortho* methyl substituents are present in the diamine used for polyimide synthesis. DPPD-MBDAM polyimide showed the best gas productivity values with 565 barrer CO_2_ permeability and a selectivity of 16 for CO_2_/CH_4_.

## Abstract 2

Three new polyimides were synthesized from 3,8-diphenylpyrene-1,2,6,7-tetracarboxylic dianhydride (DPPD) with two diamines with *ortho* methyl substitution (MBDAM and HFI) and one diamine without *ortho* substituents (BAPHF).

## Graphical abstract

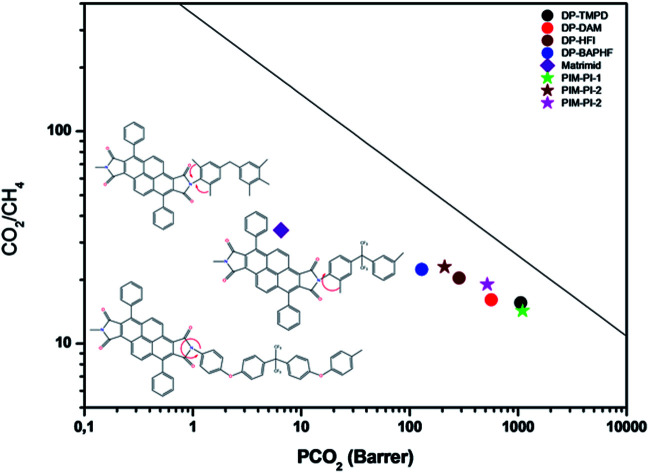

## Introduction

Aromatic polyimides continue to be the subject of extensive research in the field of membrane technology, particularly for gas separation, due to their high separation efficiency, excellent thermal stability, chemical resistance and mechanical properties.^1–3^ However, there still are some challenges in the development of new materials with high gas separation performance. For instance, several strategies based on structure-property relationships have been evaluated, such as the polymerization of monomers with bulky pendant groups which offer the possibility to achieve high free volume polymers.^4^

The most commonly used dianhydride monomers in the preparation of polyimides for gas separation membranes are 4,4′-(hexafluoroisopropylidene)diphthalic anhydride (6FDA), pyromellitic dianhydride (PMDA) and 4,4′-oxidiphthalic anhydride (ODPA).^5–10^ Nevertheless, the synthesis of new dianhydrides is a subject of considerable current interest since they allow the design and synthesis of novel polyimides for gas separation membranes. In this regard, Ma *et al.* reported two novel carbocyclic pseudo-Tröger's derived dianhydrides, which were employed to prepare soluble polyimides with an excellent gas separation performance, exceeding the upper bound for O_2_/N_2_, and H_2_/CH_4_.^11^ Ma and Pinnau, reported the synthesis of polyimides synthetized with two new ethanoanthracene-based dianhydrides that exhibited good performance for gas separation in particular for H_2_/CH_4_ and O_2_/N_2_ separation.^12^ Santiago-García *et al.* reported the synthesis of three polyimides based on 3,8-diphenylpyrene-1,2,6,7-tetracarboxylic dianhydride (DPPD). DPPD with a rigid structure and bulky groups showed to be an excellent candidate for polyimides synthesis for gas separation membranes due to an excellent combination of permeability and selectivity.^13^ However, in order to understand the full potential of this pyrene dianhydride structure for performing gas separation, further studies on DPPD polyimides are necessary to develop new polyimides, that allow us to understand how the use this dianhydride DPPD or similar structures could enhance polyimide membrane properties and separation performance.

It was reported that better performing polymers for membrane application would be obtained by increasing polymer chain rigidity but maintaining the gas diffusivity by increasing interchain separation.^4,14^ The use methyl groups have been reported as molecular spacers, increasing interchain separation which will allow increases in gas permeability by enhancing fractional free volume. Tanaka *et al.*, synthetized polyimides and reported an increases in fractional free volume and permeability, with *ortho* methyl substitution on the diamine phenyl ring.^15^ Naguel *et al.* have reported about the effects *ortho* methyl groups on polyimide and poly(amide-imide) on free volume and gas permeation. The introduction of *ortho* methyl groups increases the average *d*-spacing and the gas permeability.^16^

Therefore, in this work, we report the synthesis of three novel aromatic polyimides based on the DPPD dianhydride using a di-*ortho* methyl substituted diamine, 4-4′-methylenbis(2,6-dimethyl-aniline) (MBDAM), a mono methyl substituted diamine, 5,5′-(hexafluoroisopropylidene)-di-*o*-toluidine) (HFI), and a non-methyl substituted diamine, 2,2-bis[4-(4-aminophenoxy)phenyl] hexafluoropropane (BAPHF). Hence, we are trying to elucidate the effect of the use of aromatic diamines with and without *ortho* substituted methyl groups linked to the imide bond. The differences in polyimide solubility, mechanical properties under tension, thermal properties, physical properties and gas separation performance are determined and the changes due to the *ortho* methyl substitution discussed.

## Experimental

### Materials

Solvents and reagents were purchased from Sigma Aldrich. Nitrobenzene (NB), pyridine (Py), benzoic acid, 4-4′-methylenbis(2,6-dimethyl-aniline) (MBDAM) and 5,5′-(hexafluoroisopropylidene)-di-*o*-toluidine) (HFI) were used as received. 2,2-Bis[4-(4-aminophenoxy)phenyl] hexafluoropropane (BAPHF) was purified by crystallization in a solution of ethanol/water (70/30 v/v). 3,8-Diphenylpyrene-1,2,6,7-tetracarboxylic dianhydride (DPPD) was synthesized through a multistep synthetic route according to the procedure that has been previously reported and crystallized in nitrobenzene before use.^13^

### Polymer synthesis

**Fig. 1:**
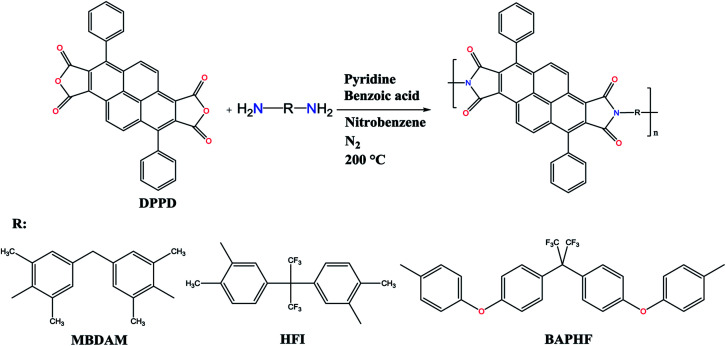
Schematic synthesis of DPPD based polyimides.

The polyimides were synthesized *via* polycondensation reaction from the base dianhydride DPPD and three aromatic diamines (MBDAM, HFI, BAPHF), using the one-step method as shown in Fig. 1. In a typical synthesis, DPPD-MBDAM was prepared according to the following procedure. 1 mmol MBDAM was dissolved in 6 mL of nitrobenzene in a 50 mL three-neck flask equipped with a nitrogen inlet/outlet, and a mechanical stirrer. 1 mmol of DPPD was added and the mixture was heated to 80 °C. Then, 2 mmol of pyridine were added and the solution was maintained at that temperature for 1 h. The reaction solution was heated to 120 °C and 2 mmol of benzoic acid were added. The solution was heated to 200 °C for 24 h. After cooling, the viscous solution was poured into ethanol to obtain a yellow fibrous polymer. This material was filtered off, washed with ethanol and dried under vacuum at 200 °C for 24 h. The yield of this polyimide was 93%. Polyimides DPPD-HFI and DPPD-BAPHF were synthesized by a similar procedure.

### Film preparation

Dense films were prepared by a casting method using the following procedure. Each polyimide was dissolved in nitrobenzene a 4% (w/v). The polyimide solution was filtered through a 200 μm Teflon syringe filter and then poured onto an aluminum ring placed on a glass plate. Subsequently, the solvent was evaporated at 80 °C for 12 h. The film was dried under vacuum at 230 °C for 48 h. The complete removal of solvent was confirmed using thermogravimetric analysis.

### Characterization

Solubility was determined by dissolving 5 mg of polymer in 1 mL of solvent at room temperature. ^1^H NMR spectra were recorded on a Varian VNMRS 600 MHz spectrometer using CDCl_3_ as a solvent. Fourier transform infrared (FTIR) spectra were recorded on a Nicolet 8700 Thermo Scientific FTIR spectrometer using an ATR accessory.

Inherent viscosities (*η*_inh_) were measured using an Ubbelohde viscometer at a polyimide concentration of 0.5 g dL^−1^ in DMF at 30 °C. For MBDAM*η*_inh_ was determined in nitrobenzene at 50 °C. Thermogravimetric analyses (TGA) were performed on a thermobalance TGA 7 under nitrogen atmosphere at a heating rate of 10 °C min^−1^. Differential scanning calorimetry (DSC) measurements were carried out using a Mettler-Toledo DSC 1 Star System at a heating rate of 10 °C min^−1^ under nitrogen atmosphere. Mechanical properties under uniaxial tension were determined on a Shimadzu AGS-X universal testing machine with a 100 N load cell at a crosshead speed of 1 mm min^−1^. Films with dimensions of 0.5 cm long × 2 cm wide and thicknesses of 100 μm (DPPD-MBDAM and DPPD-HFI) and 60 μm (DPPD-BAPHF) were used. At least five individual samples were tested for each polyimide film.

X-ray diffraction (XRD) was conducted on a Bruker D8 Advance diffractometer with CuKα radiation (wavelength *λ*_Cu_ = 1.542 Å), in range of 5° to 60° 2*θ*. The average *d*-spacing was calculated using Bragg's law:
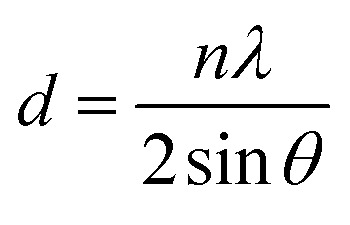
where *θ* of the broad peak maximum. Polyimide density (*ρ*) was measured in a density gradient column (Techne Corporation) with calcium nitrate solutions at 23 °C. The fractional free volume (FFV) was calculated from the density data using the following relation:
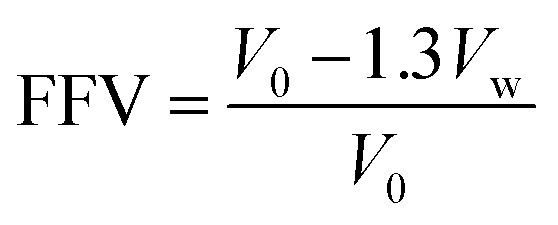
where *V* is specific volume of the polymer (*i.e.*, the inverse of the polymer density) and *V*_w_ is the van der Waals volume, which was estimated by the group contribution method outlined by Zhao *et al.*^17^

Pure gas permeability coefficients (*P*) were determined using a constant volume permeation cell of the type described elsewhere,^18^ according to the following equation:
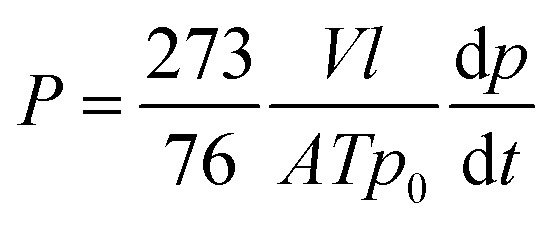
where *A* and *l* are, the effective area and the thickness of the film, *T* is the temperature of the measurement (308.15 K), *V* is the constant volume of the permeation cell, *p*_0_ is the pressure of the feed gas in the upstream and d*p*/d*t* is the gas pressure increase with time under steady state conditions measured in the permeation cell. *P* is expressed in Barrer [1 barrer = 10^−10^ cm^3^ (STP) cm cm^−2^ s^−1^ cm Hg^−1^]. The apparent diffusion coefficient, *D*, was obtained by the time lag method using the relation:
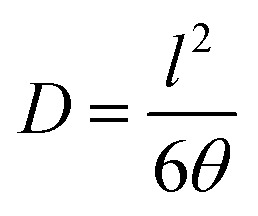
here *l* is the film thickness and *θ* is the time lag. The apparent solubility coefficient, *S*, was obtained using the relationship
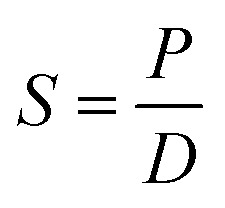


## Result and discussion

**Fig. 2:**
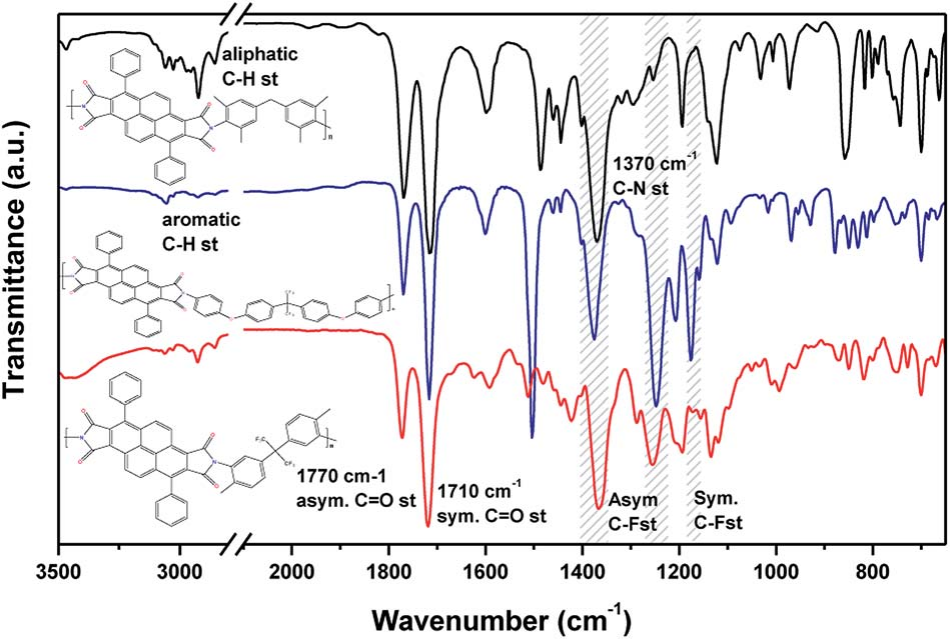
FTIR spectra of polyimide films.

**Fig. 3:**
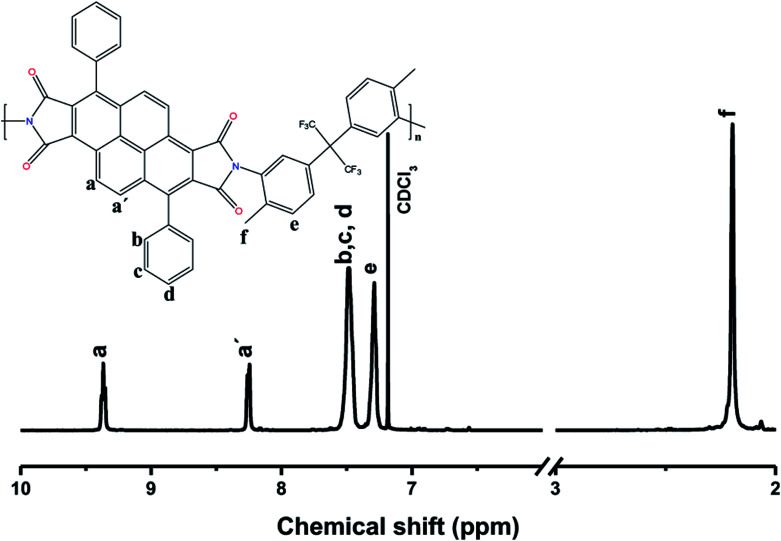
^1^H NMR spectra of the polyimide DPPD-HFI.

Three new polyimides were synthesized by one-step high-temperature polycondensation reaction between the previously synthesized 3,8-diphenylpyrene-1,2,6,7-tetracarboxylic dianhydride (DPPD) and commercial diamines, using nitrobenzene as solvent, and pyridine and benzoic acid as shown in Fig. 1. Fig. 2 shows the ATR-FTIR spectra of the three polyimides studied. Characteristic absorption bands of polyimides appeared at 1770 and 1710 cm^−1^ (C=O asymmetrical and symmetrical stretching), 1370 cm^−1^ (C–N stretching). For substituted polyimides, typical aliphatic C–H absorption bands at 2940–2860 cm^−1^ were also observed from those with –CH_3_ substitution (DPPD-MBDAM and DPPD-HFI). Also, DPPD-BAPHF spectrum shows characteristic bands 1170 and 1207 cm^−1^ (–CF_3_ symmetrical and asymmetric stretching). The fully imidized structure is confirmed by the absence of absorption bands in 3300 cm^−1^ (C–N stretching) present in the non-cyclized structure of the corresponding poly(amic acid). DPPD-MBDAM, DPPD-HFI and DPPD-BAPHF polyimides were also characterized by ^1^H-NMR spectroscopy. As an example, Fig. 3 shows the ^1^H-NMR spectra of polyimide DPPD-HFI, in which all the protons in the structure are assigned. The signals assigned to the protons of methyl groups were detected at 2.19 ppm. The aromatic protons resonated in the region of 7.30–9.40 ppm. The spectra confirm the imidized structure, as indicated by the absence of poly(amic acid) protons.

Polyimides solubility was evaluated dissolving 5 mg of polymer in 1 mL of solvent. All polyimides except DPPD-BAPHF were soluble in CHCl_3_ at ambient temperature and insoluble in DMSO, as shown in Table 1.

**Table 1:** Solubility and viscosity of DPPD based polyimides

Polyimide	Solubility							*η*_inh_ (dL g^−1^)
	CHCl_3_	DMAc	DMF	THF	NMP	DMSO	NB	
DPPD-MBDAM^a^	+	−	−	−	−	−	*+	0.79
DPPD-HFI^b^	+	+	+	+	+	−	+	0.32
DPPD-BAPHF	−	−	−	−	−	−	±	−

a *η*_inh_ measured in nitrobenzene at 50 °C.

b *η*_inh_ measured in DMF at 30 °C. +: soluble; −: insoluble; *+: soluble on heating at boiling point; ±: partially soluble on heating at boiling point. NMP: 1-methyl-2-pyrrolidone; DMF: *N*,*N*-dimethylformamide; DMAC: *N*,*N*-dimethylacetamide; DMSO: dimethyl sulfoxide; THF: tetrahydrofuran; NB: nitrobenzene.

However, DPPD-HFI exhibited better solubility, in both polar aprotic solvents such as DMAc, DMF, DMSO and NMP and low boiling point solvents, CHCl_3_ and THF. Moreover, DPPD-BAPHF showed the lowest solubility since it is partially soluble in nitrobenzene on heating at the boiling point. Based on these differences in solubility, nitrobenzene was used for casting membranes in order to maintain the same thermal history. Also Table 1 shows the inherent viscosities (*η*_inh_) of DPPD based polyimides. *η*_inh_'s of DPPD polyimides were in the range of 0.32 dL g^−1^ to 0.79 dL g^−1^, which are related to moderate to high molecular weight polymers similar to some structurally rigid polyimides reported in the literature.^19,20^ In the case of DPPD-BAPFH which is not soluble under the test conditions intrinsic viscosity was not determined.

### Thermal properties

**Fig. 4:**
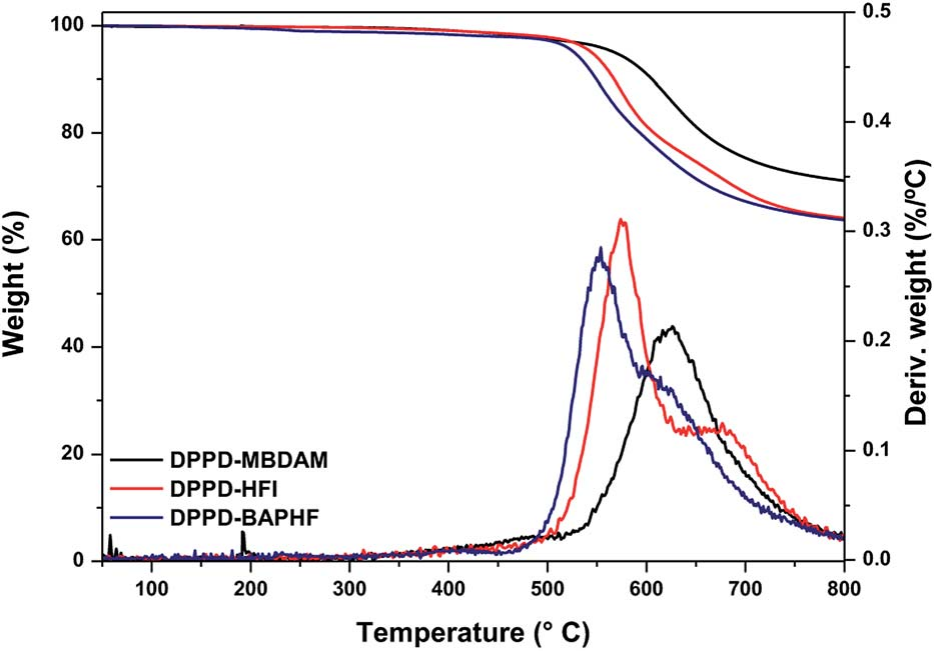
TGA thermograms of the DPPD based polyimides.

The thermal properties of the DPPD based polyimides were evaluated by TGA and DSC and the results are summarized in Table 2. The glass transition temperature (*T*_g_) of these polyimides was in the range of 336 to 369 °C and decreased in the following order DPPD-MBDAM > DPPD-HFI > DPPD-BAPHF. The DPPD-MBDAM with two *ortho* methyl substitutions close to the –C–N– link in the imide ring had the highest *T*_g_ value at 369 °C, while DPPD-BAPHF without substituent in the *ortho* position of the BAPHF diamine phenyl bonded at the imide has the lowest *T*_g_ value at 338 °C. It is well know that the glass transition is affected by the chain conformation (rigidity and linearity) and interchain interactions.^2,21^ The *ortho* methyl substituted DPPD-MBDAM and DPPD-HFI may restricts the rotation of the C–N bond in the polyimide that results in a higher chain rigidity and increase the inter-chain interaction due the electro-donating nature of the CH_3_ group, which resulted in an increase in *T*_g_.^2^ Therefore, the presence of two –CH_3_ in the *ortho* phenyl position induces a *T*_g_ increase in DPPD-MBDAM as compared to DPPD-HFI. While DPPD-BAPHF showed the lowest *T*_g_ value due to a larger degree of conformational freedom attributed to the presence of the ether linkages in the main polymer chain. Thermal stability thermograms for DPPD-MBDAM, DPPD-HFI and DPPD-BAPHF are shown in Fig. 4 and thermal decomposition data are reported in Table 2. The 5% (*T*_5_) and 10% (*T*_10_) weight losses were found to occur at temperature ranges from 526 to 567 °C and 548 to 605 °C respectively. All polyimides show high thermal stability in nitrogen atmosphere, with a degradation temperature (*T*_d_) above 515 °C.

**Table 2:** Thermal properties of the DPPD based polyimides^a^

Polymer	*T*_d_ (°C)	*T*_5_ (°C)	*T*_10_ (°C)	Char yield^b^ (%)	*T*_g_
DPPD-MBDAM	546	567	605	71	369
DPPD-HFI	517	545	568	64	352
DPPD-BAPFH	515	526	548	63	338

a *T*_d_: degradation temperature measured in onset, *T*_5_ y *T*_10_: degradation temperature at 5 y 10% weight loss, respectively.

b Measured at 800 °C in N_2_.

The high char yield of these polyimides was due to the high aromatic content of the polymers. Moreover, DTGA curves of DPPD-BAPHF and DPPD-HFI show two degradation steps under nitrogen. The first weight loss, around 500 °C, is attributed to the –CF_3_ group degradation, whereas the second weight loss occurring around 600 °C could be attributed to main chain decomposition.

### Mechanical properties

Table 3 show mechanical properties of DPPD based polyimides; they showed Young's modulus and tensile strengths in the range of 1.20–1.30 GPa and 42–62 MPa, respectively. The three polyimide films presented similar Young modules, around 1.2 GPa. DPPD-HFI, show the lowest elongation at break and tensile strength due to a lower inherent viscosity. DPPD-MBDAM exhibited the highest tensile strength at 62 MPa and tensile modulus 1.29 GPa. These mechanical properties are comparable to other highly aromatic polyimides previously reported in the literature.^20,22,23^

**Table 3:** Mechanical properties of DPPD based polyimide membranes

Polymer	Young's modulus (GPa)	Tensile strength (MPa)	Elongation at break (%)
DPPD-MBDAM	1.29 ± 0.01	62 ± 6	7.3 ± 2
DPPD-HFI	1.27 ± 0.04	42 ± 2	4 ± 1
DPPD-BAPHF	1.20 ± 0.05	53 ± 5	8 ± 2

**Fig. 5:**
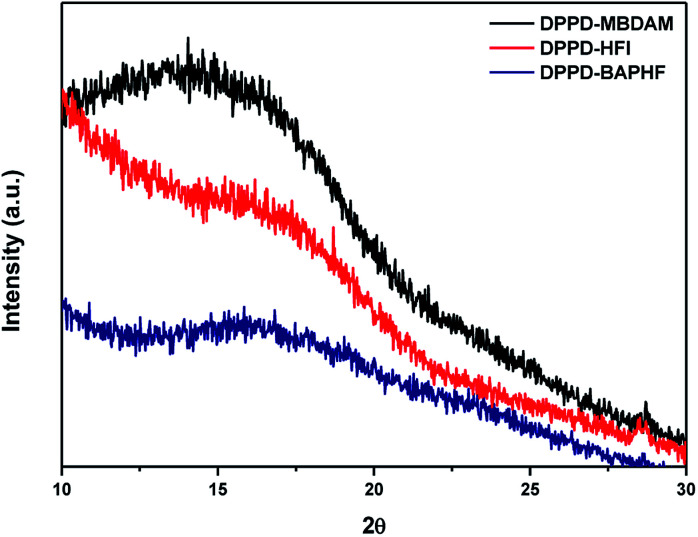
X-ray diffraction pattern for DPPD based polyimides.

Fig. 5 shows X-ray diffraction (XRD) patterns for DPPD based polyimides. The resulting patterns revealed that all the polyimides are amorphous with broad peaks. The average interchain distances (*d*-spacing) values were calculated from the position of the maximum in the broad X-ray diffraction pattern. *d*-spacing is often related to the average chain distance and packing density of polymer chains.^24^ X-ray diffraction pattern for DPPD-MBDAM shows a broader shoulder than DPPD-HFI and DPPD-BAPHF. The maximum of this shoulder corresponds to a *d*-spacing of 6.1 Å (2*θ* = 14.49). DPPD-HFI shows 2 maxima in the amorphous halo corresponding to a 5.33 Å (2*θ* = 16.80) and 8.807 Å *d*-spacing (2*θ* = 10.01). While DPPD-BAPHF show one maxima with *d*-spacing at 5.21 Å (2*θ* = 16.80). The *ortho* substitution in MBDAM restricts the mobility of the polymer chains and increase the interchain distance, this effect can observed in the position and shape of the amorphous halo that agrees with the results found. Also, these results are in agreement with fractional free volume FFV values, as show in Table 4. According to the literature, polymers with larger *d*-spacing generally tend to have a larger FFV.^25^ FFV shows a decrease in the following order DPPD-MBDAM > DPPD-HFI > DPPD-BAPHF. For DPPD based polyimides containing methyl in the *ortho* position of the diamine the internal rotation around the imide bond is restricted between the phenyl ring and the imide which inhibits chain packing. As a result they present higher fractional free volume as in this case does DPPD-MBDAM and DPPD-HFI as compared to DPPD-BAPHF.

### DPPD based polyimides gas transport properties

Pure gas permeability coefficients, *P*, for H_2_, He, O_2_, N_2_, CH_4_ and CO_2_ and the ideal selectivity for important gas pairs O_2_/N_2_, CO_2_/CH_4_ and CO_2_/N_2_, are reported in Table 5. The permeability coefficients order found is CO_2_ > H_2_ > He > O_2_ > CH_4_ > N_2_, where *P* of N_2_ (3.64 Å) is lower than the one of CH_4_ (3.8 Å), and CO_2_ (3.3 Å) shows a *P* higher than He (2.6 Å). This is attributed to the high FFV presented in DPPD polyimides and permeability is comparable to the values reported for other polyimides of high FFV.^3,4,24^DPPD polyimides show excellent gas permeability coefficients for the six gases tested and moderate selectivities that follows the order DPPD-MBDAM > DPPD-HFI > DPPD-BAPHF. This result is attributed to the presence of methyl groups in *ortho*-position nearby the –C–N– imide bonds that lower the local mobility of the polyimide chains, which is reflected in an increase of gas permeability.^13,21,26^ This increase in gas permeability is higher for the disubstituted methyl polyimides. For example, the presence of two –CH_3_ on MBDAM, leads to a difference between 2 and 6 times in gas permeability coefficients for the ratio *P*_(__MBDAM__)_/*P*_(__BAPHF__)_, which is higher for the most condensable gases CO_2_ and CH_4_. Moreover, the presence of one –CH_3_ on HFI, leads to a 2 fold increase in permeability for the ratio *P*_(__HFI__)_/*P*_(__BAPHF__)_. This effect is in agreement with previous reports, which indicate that the introduction of *ortho* methyl substituted groups in the diamine moieties can increase gas permeability coefficients but they follow the usual trade off since there is a decreased in selectivity.^27,28^

**Table 4:** Physical properties of DPPD based polyimides

Polymer	*d*_1_ spacing (Å)	Density (g cm^−3^)	*V*_dw_ (cm^3^ mol^−1^)	FFV
DPPD-MBDAM	6.23	1.181	384.03	0.170
DPPD-HFI	5.31	1.30	405.96	0.164
DPPD-BAPHF	5.09	1.32	483.15	0.152

Apparent gas diffusion coefficients, *D*, determined from time lag and apparent gas solubility coefficient *S*, (calculated from *D* and *P*), are presented in Table 6. Also the values of diffusivity selectivity, *α*(*D*_A_/*D*_B_), and solubility selectivity. *α*(*S*_A_/*S*_B_), are included. Table 6 shows that the introduction of methyl groups into the *ortho* position of polyimides increase the diffusion and solubility coefficients for all gases. This increase is larger for polyimides with *ortho* methyl groups. For example, the diffusion coefficients for DPPD-MBDAM exhibited 2–3 times those found for DPPD-BAPHF and 1.2–1.5 times the solubility coefficient. DPPD-MBDAM presents 1.2–1.6 fold higher diffusion coefficient and 1.5–2 solubility coefficient as compared to DPPD-HFI. Meanwhile, DPPD-HFI presents 2 fold higher diffusion coefficients and 1.2 fold higher solubility coefficients relative to DPPD-BAPHF. Moreover, Table 6 show that the increase in solubility of *S*_(__MBDAM__)_/*S*_(__HFI__)_ is higher than *S*_(__HFI__)_/*S*_(__BAPHF__)_; although in both cases the difference is the presence of one methyl group. This can be attributed to a decrease in solubility of the polyimides with the presences of CF_3_ groups.

**Table 5:** Gas permeability coefficients and ideal selectivity at 2 atm and 35 °C for DPPD based polyimides^a^

Polymer	Permeability (barrer)							Selectivity (*α*_A/B_)	
	*P*_He_	*P*_H_2__	*P*_O_2__	*P*_N_2__	*P*_CH_4__	*P*_CO_2__	*α*_O_2_/N_2__	*α*_CO_2_/CH_4__	*α*_C0_2_/N_2__
DPPD-MBDAM	177	349	79	23	35	565	3.4	16.1	24.5
DPPD-HFI	174	257	50	14	14	286	3.5	20.4	20.4
DPPD-BAPFH	87	113	20	5.34	5.74	129	3.7	22.4	24.2

a 1 barrer = 10^−10^ cm^3^ (STP) cm cm^−2^ s^−1^ cm Hg^−1^.

Apparent gas diffusion coefficients for DPPD based polyimides

**Table:** 

Polymer	*D* (10^−8^ cm^2^ s^−1^)				*α*(*D*_A_/*D*_B_)		
	*D*_O_2__	*D*_N_2__	*D*_CH_4__	*D*_CO_2__	*α*_O_2_/N_2__	*α*_CO_2_/CH_4__	*α*_CO_2_/N_2__
DPPD-MBDAM	28.3	9.2	3.4	15	3.1	4.5	1.7
DPPD-HFI	23.0	7.5	2.1	11.6	3.1	5.5	1.5
DPPD-BAPFH	10.8	3.5	1.1	6.4	3.1	5.9	1.8

**Table:** 

Polymer	*S* (10^−2^ cm^3^ (STP) cm^−3^ cm Hg^−1^)				*α*(*S*_A_/*S*_B_)		
	*S*_O_2__	*S*_N_2__	*S*_CH_4__	*S*_CO_2__	*α*_O_2_/N_2__	*α*_CO_2_/CH_4__	*α*_CO_2_/N_2__
DPPD-MBDAM	2.8	2.5	10.3	36.8	1.1	3.6	14.5
DPPD-HFI	2.2	1.9	6.6	24.7	1.2	3.7	13.1
DPPD-BAPFH	1.8	1.5	5.3	20.2	1.2	3.8	13.3

**Fig. 6:**
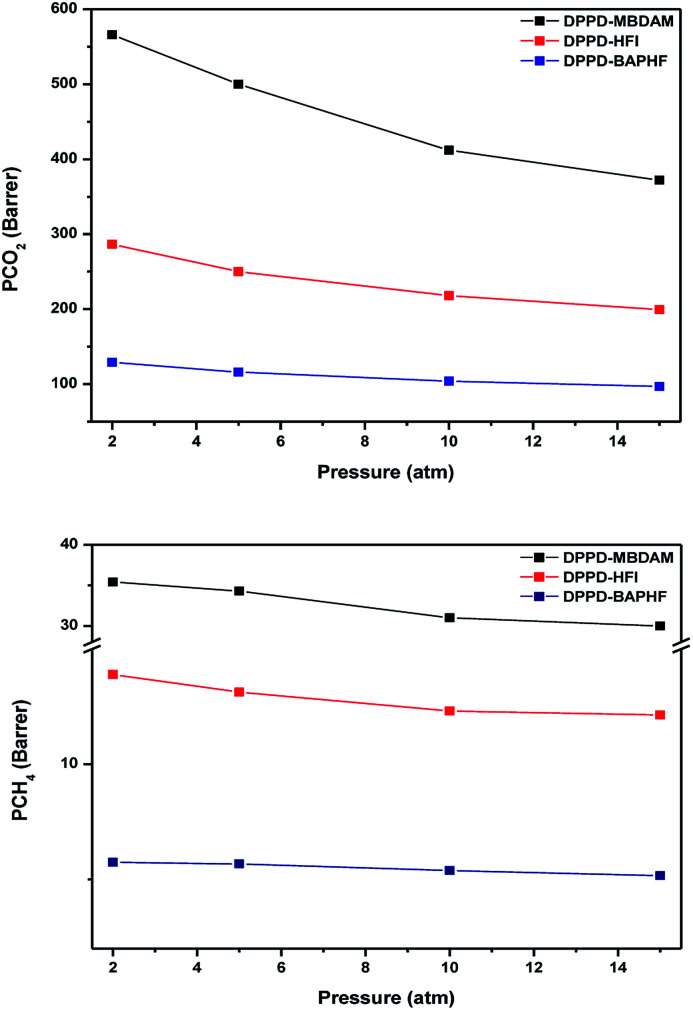
Pressure dependence of pure CO_2_ and CH_4_ permeabilities for DPPD based polyimides.

Plasticization is a phenomenon that has significant practical implications in membrane for gas separation particularly for CO_2_ removal from natural gas.^29,30^ Plasticization often results in an increase in permeability and a loss in selectivity, particularly at high pressures.^31^ To determine any potential plasticization effects f on DPPD-MBDAM, DPPD-HFI and DPPD-BAPHF membranes, pure gas CO_2_ and CH_4_ permeabilities were also measured at 35 °C from 2 to 15 atm (Fig. 6). As it is usually found in other polymers, pure CO_2_ and CH_4_ permeability coefficients of these polyimides decreased with increasing feed pressure up to 15 atm.^27,29,32,33^ This preliminarily results indicates that DPPD-MBDAM, DPPD-HFI and DPPD-BAPHF membranes do not present plasticization up to a testing pressure of 15 atm.

**Fig. 7:**
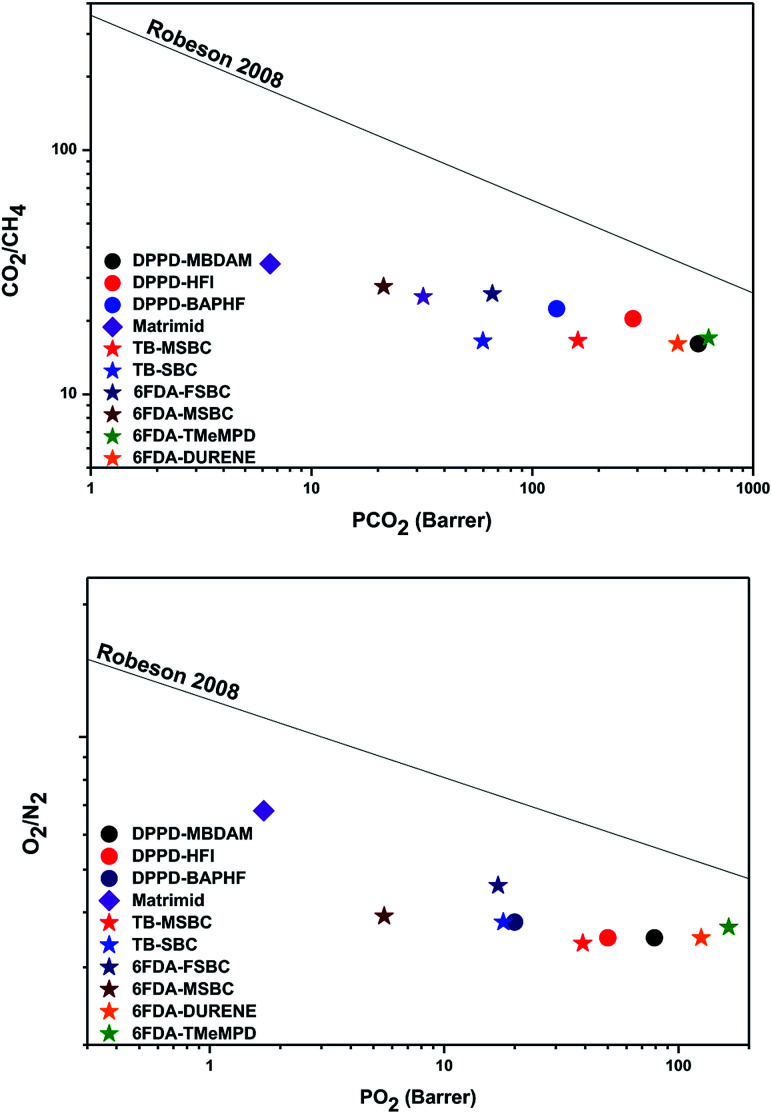
CO_2_/CH_4_ and O_2_/N_2_ productivities for DPPD based polyimides.

Fig. 7 compares the productivities of the synthesized polyimides with Matrimid®^34^ and polymers with different group substitutions recentl*y* published^2,21^ in a Robeson upper bound plot.^35^ From these plots it can be observed that for the pairs CO_2_/CH_4_ and O_2_/N_2_, the polyimides based on the DPPD dianhydride and the different diamines lead to permeability-selectivity performance that is closer to the upper bound. DPPD based polyimides show a combination of high gas permeability coefficients and moderate selectivity. Therefore, the *ortho* methyl substituted diamines combined with DPPD leads to polyimides with a positive effect on the gas separation performance compared to polymers with different substitutions based in spirobichroman and Trögers base^2,21^ and similar effect with 6FDA based polyimides.

## Conclusions

Three new polyimides were synthesized from the dianhydride DPPD*via* one-step high-temperature polycondensation. These polyimides exhibited high thermal stability with decomposition temperatures above 505 °C and *T*_g_ above 338 °C. DPPD-MBDAM and DPPD-HFI polyimides show a relatively higher *d*-spacing and FFV due to the introduction of the methyl group into the *ortho* position of the diamine linked to de imide (–C–N) which decrease the chain packing and increases FFV and *d*-spacing. The *ortho* methyl substitution in the used diamine improves gas permeability coefficients with small decreases in gas pair selectivity for these polyimides. Moreover, the *ortho* methyl substitution and the number of *ortho* methyl groups present induce an increase in gas permeability with a decrease in selectivity. In particular, the DPPD-MBDAM shows the largest gas permeability with *P*_CO_2__ = 565 and selectivity CO_2_/CH_4_ = 16, with two *ortho* methyl substitutions close to the imide linkage.

## Conflicts of interest

There are no conflicts of interest to declare.

## Supplementary Material
